# Severe mental illness and quality of diabetes care: a retrospective national linked cohort study.

**DOI:** 10.23889/ijpds.v7i3.1987

**Published:** 2022-08-25

**Authors:** Stine Scheuer, Kelly Fleetwood, Kirsty Licence, Stewart Mercer, Daniel Smith, Sarah Wild, Caroline Jackson

**Affiliations:** 1 Steno Diabetes Center; 2 University of Edinburgh; 3 National Services Scotland, NHS Scotland

## Objective

To use a Scottish national linked diabetes care dataset to determine whether annual monitoring of clinical guideline-recommended diabetes care indicators differ by severe mental illness (SMI) status among people with type 2 diabetes (T2D) during the first year following diabetes diagnosis and thereafter.

## Approach

We examined receipt of 9 diabetes care indicators (HbA1c; cholesterol; urinary albumin; serum creatinine; blood pressure; retinopathy screening; foot examination; body mass index; and smoking status) in people with T2D diagnosed 2009-2018, comparing people with a prior hospital record of schizophrenia, bipolar disorder and depression versus no hospital record for any mental illness. We analysed receipt of care in the first year using logistic regression and for the entire follow-up period (until death or 31 December 2018) using generalized linear mixed effects models, adjusting for calendar year, sociodemographic and lifestyle factors and comorbidities (and in the latter, diabetes duration).

## Results

We included 119,508 people with T2D (mean age 59.7 [±13.2 SD]), of which 1701 (1%), 768 (0.5%) and 5211 (3%) had schizophrenia, bipolar disorder and depression, respectively. During the first year, compared to people without mental illness those with each SMI were more likely to receive cholesterol and serum creatinine measurement and, for schizophrenia only, HbA1c measurement, after adjustment for confounding factors. People with depression had higher odds of receiving a smoking review, but lower odds of receiving urinary albumin measurement. Receipt of all other indicators were similar across comparison groups. In the longitudinal analyses, people with schizophrenia had persistently higher odds of receiving HbA1c and people with a SMI had lower odds of receiving retinopathy screening.

## Conclusion

Receipt of care delivered in the primary care setting was generally similar in people with versus without SMI in the short and long term. However, people with SMI were less likely to receive retinopathy screening, highlighting a need for better monitoring of care indicators delivered outside primary care settings.

